# No DNA damage response and negligible genome-wide transcriptional changes in human embryonic stem cells exposed to terahertz radiation

**DOI:** 10.1038/srep07749

**Published:** 2015-01-13

**Authors:** A. N. Bogomazova, E. M. Vassina, T. N. Goryachkovskaya, V. M. Popik, A. S. Sokolov, N. A. Kolchanov, M. A. Lagarkova, S. L. Kiselev, S. E. Peltek

**Affiliations:** 1 Vavilov Institute of General Genetics RAS, Moscow, Russia; 2 Institute of Cytology and Genetics RAS, Novosibirsk, Russia; 3 Budker Institute of Nucleic Physics SB RAS, Novosibirsk, Russia; 4 Centre “Bioengineering” RAS, Moscow, Russia; 5 Scientific Research Institute of Physical-Chemical Medicine, Moscow, Russia; 6 Skoltech Center for Stem Cell Research, Skolkovo Institute of Science and Technology, Skolkovo, Moscow Region, Russia

## Abstract

Terahertz (THz) radiation was proposed recently for use in various applications, including medical imaging and security scanners. However, there are concerns regarding the possible biological effects of non-ionising electromagnetic radiation in the THz range on cells. Human embryonic stem cells (hESCs) are extremely sensitive to environmental stimuli, and we therefore utilised this cell model to investigate the non-thermal effects of THz irradiation. We studied DNA damage and transcriptome responses in hESCs exposed to narrow-band THz radiation (2.3 THz) under strict temperature control. The transcription of approximately 1% of genes was subtly increased following THz irradiation. Functional annotation enrichment analysis of differentially expressed genes revealed 15 functional classes, which were mostly related to mitochondria. Terahertz irradiation did not induce the formation of γH2AX foci or structural chromosomal aberrations in hESCs. We did not observe any effect on the mitotic index or morphology of the hESCs following THz exposure.

New sources of non-ionising terahertz (THz) radiation are rapidly emerging due to recent progress in laser and semiconductor technologies. THz technology has been introduced into many practical and research applications in medicine, defence, and security^1^. Exposure of humans to THz radiation is expected to increase, although relatively little is known about the effects of this radiation on biological systems. THz radiation is non-ionising electromagnetic radiation with a wavelength of 30–3000 μm. The energy of THz radiation is not sufficient to cause ionisation in DNA or other biological materials. Therefore, it is not surprising that the majority of genotoxic studies did not reveal any effect of THz radiation on the structure of DNA^2^,^3^. The main impact of THz radiation on biological systems is a thermal effect due to the high absorption of THz radiation by water^1^. However, four decades ago, Fröhlich assumed that THz radiation also has a non-thermal (or microthermal) effect mediated by the excitation of specific biological macromolecules or linear/nonlinear resonance mechanisms^4^. Recently, a mathematical model of DNA breathing was suggested^5^. This model predicted that THz radiation creates a local opening in the DNA helix through nonlinear resonance and can thereby influence gene expression and DNA replication. A subsequent theoretical study confirmed the existence of destabilising DNA breather modes, although DNA denaturation under THz exposure is extremely unlikely *in vivo* because of the predominating effect of thermal noise^6^. The data obtained in a study of mouse mesenchymal stem cells (MSCs) that responded to THz irradiation by adjusting the expression of specific genes might be explained by the DNA breathing model^7^. Importantly, computer simulations have demonstrated intrinsic DNA breathing dynamics in the core promoters of genes that are susceptible to THz exposure. The non-thermal effect of THz radiation on gene expression was also observed in a study of artificial human skin^8^. Both investigations revealed that THz induces changes in the expression of genes implicated in differentiation. This raises the question of whether THz radiation influences the properties of stem cells, especially the fragile balance between self-renewal and differentiation. Additionally, it was proposed that THz radiation can be a potential tool for differentiation of stem cells^9^. Pluripotent stem cells represent a unique natural type of “universal” stem cells, and their differentiation capabilities driven by THz irradiation may represent a very promising approach to their practical application.

Stem cells are capable of unlimited self-renewal and have an intrinsic ability to differentiate into specialised cells. Interference with these essential properties of stem cells can lead to developmental disorders and tissue depletion. Stem cells cultivated *in vitro* provide a means to investigate developmental toxicity as well as cytotoxic and genotoxic effects. Alexandrov *et al*.^7^ and Bock et al.^9^ studied the capacity of THz radiation to affect the differentiation of mouse MSCs. They reported that THz irradiation accelerates the differentiation of MSCs into adipocytes by altering transcription. This effect depends on the duration of exposure and the frequency of THz radiation as well as on the stage of MSC differentiation. Another investigation involving human embryonic stem cells (hESCs) which did not reveal any effect of THz radiation on the proliferation, morphological properties and pluripotency maintenance of these cells^10^. However, this previous study did not include genome-wide transcriptome analysis.

Most previously published studies of biological effects of THz irradiation on mammalian cell lines (tumour cell lines or primary cell culture) did not report serious DNA damage upon exposure to THz^2^. Therefore, we decided to use pluripotent stem cells as a cell type that is extremely sensitive to culture conditions and DNA damage reacting immediately with transcriptional changes, spontaneous differentiation and apoptosis^11^,^12^,^13^. Thus, embryonic stem cells would be the appropriate choice to study the influence of physical processes on DNA integrity and cell machinery.

The main aim of the current study was to explore the non-thermal effects of narrow-band THz radiation on transcription in hESCs. We also examined chromosome aberrations and γH2AX foci in hESCs, as well as their mitotic index, following THz exposure. Irradiated and control hESCs did not differ in terms of these three genotoxic endpoints. Nevertheless, transcriptome analysis allowed us to identify a limited set of genes that responded to THz radiation and that shared common characteristics.

## Results

### The influence of THz radiation on hESC morphology

**Figure 1:**
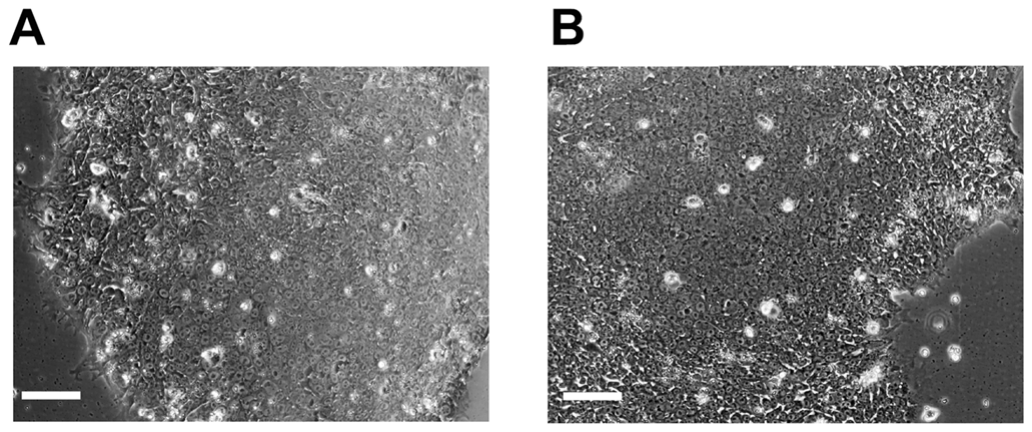
Human embryonic stem cells retain their morphology after exposure to terahertz (THz) radiation. (A). A colony of control hESM01 cells. (B). A colony of hESM01 cells 20 hours after Thz exposure. Scale bar, 100 μm.

ESCs are prone to spontaneous differentiation in culture. Unfavourable conditions can lead to the rapid appearance of differentiated cells. DNA damage is able to induce differentiation of ESCs via Nanog suppression by p53^14^. We examined the morphology of irradiated hESCs 16 and 20 hours after THz exposure, and the morphological analysis did not reveal any differences between hESCs that were exposed to THz radiation and those that were not. Irradiated cells retained a high nucleus/cytoplasm ratio and continued to form round-shaped colonies characteristic of pluripotent stem cells (PSCs). No signs of spontaneous differentiation were observed (Fig. 1).

### Chromosomal aberrations after exposure to THz radiation

Cytogenetic abnormalities, particularly structural chromosomal aberrations, are among the most common markers of genotoxicity^15^. We performed cytogenetic analysis of structural chromosomal aberrations induced in hESCs by THz radiation. Irradiated and control hESCs were maintained in mTeSR medium for 20 hours prior to harvesting of the chromosome spreads. We scored chromosome-type and chromatid-type aberrations in at least 200 metaphase spreads per sample. The results of two repeat experiments were combined because the frequencies of the chromosomal anomalies did not vary significantly between the two repeats (Table 1). Cytogenetic analysis did not reveal any differences between hESCs that were exposed to THz radiation and those that were not.

### DNA double-strand breaks (DSBs) in irradiated and non-irradiated hESM01 cells

**Figure 2:**
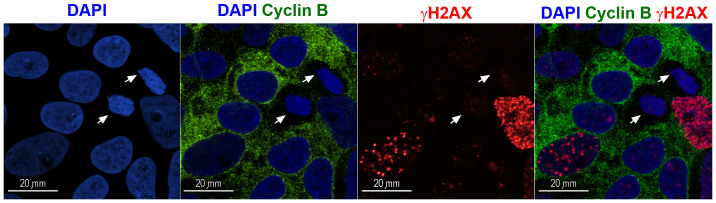
γH2AX foci (red) were scored in G1 cells, which were identified by cyclin B1 negative immunostaining (green). Nuclei stained by DAPI are shown in blue. White arrows indicate nuclei in G1 cells.

DSBs are one of the most dangerous types of DNA damage and can lead to chromosomal rearrangements and apoptosis^16^. Regardless of the mechanism underlying DSB formation, these breaks can be experimentally detected by examining the presence of phosphorylated histone H2AX. Phosphorylation of histone H2AX on serine 139 occurs shortly after DNA damage and produces distinct visible foci around DSBs (so-called γH2AX foci). γH2AX foci are a sensitive marker of DSBs and are applied widely in genotoxic studies^17^. We previously showed that the spontaneous level of γH2AX foci in hESCs is extremely variable; it depends on the cell cycle phase and is lowest in G1 cells^18^. To avoid the influence of the cell cycle, we counted γH2AX foci only in G1 cells, which were identified by negative cyclin B1 immunostaining. Figure 2 shows representative images of this staining. γH2AX foci were counted 2 hours after THz irradiation in 30–40 nuclei per sample. The number of γH2AX foci in G1 cell nuclei did not significantly differ between the irradiated and control hESCs. The mean number of γH2AX foci per nucleus was 4.1 ± 1.4 in irradiated cells and 3.7 ± 0.4 in non-irradiated cells (Table 2).

### Mitotic index of irradiated and non-irradiated hESM01 cells

**Figure 3:**
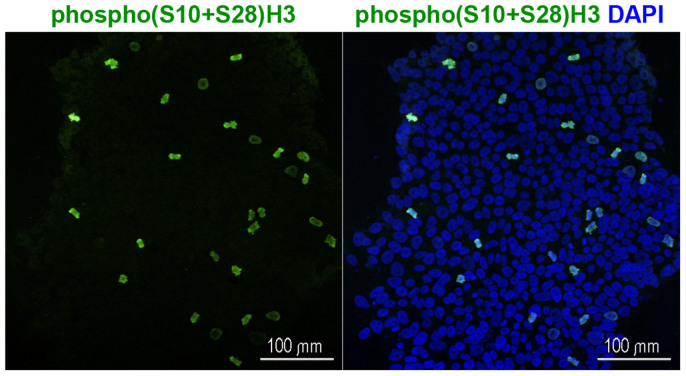
The mitotic index was analysed in irradiated and non-irradiated hESM01 cells using immunostaining with an antibody against phosphorylated (S10 and S28) histone 3 (green). Nuclei stained by DAPI are shown in blue.

Cell cycle arrest is one of the earliest cellular responses to DNA damage^19^. Cell cycle arrest at the G2/M stage leads to a sharp and long-term decrease in the number of mitotic cells after a genotoxic insult^20^. Thus, a decreased number of mitotic cells may provide indirect evidence of radiation-induced genotoxicity. To study the effects of THz radiation on the cell cycle, we counted the number of mitotic cells at 16 hours after irradiation. Mitotic cells were identified by immunocytochemical staining with an antibody against histone H3 phosphorylated at serine residues 10 and 28 (Fig. 3). We scored the number of mitotic cells in at least 5000 cells per sample. Exposure to THz radiation did not affect the frequency of mitotic cells, with a mean mitotic index of 3.2 ± 0.1% and 3.1 ± 0.4% for irradiated and control cells, respectively (Table 3).

### THz radiation enhances the transcription of genes that share common properties

Total RNA for whole genome transcriptome analysis was isolated from hESCs that were exposed to THz radiation for 1 hour and then allowed to recover for 2 hours. Gene expression was analysed using Illumina microarray technology. The raw data list consisted of 47318 transcripts, of which 15000 were detected with a p-value of <0.01 in the entire dataset. We applied a 1.5-fold change cut-off to select genes that responded to THz irradiation. The expression of 73 genes differed between THz-irradiated and non-irradiated hESM01 cells (Suppl. Table 1). In this group of genes, the mean fold change was 1.9, and the maximal fold change was 3.2. The majority of these genes (>70%) had a fold change of less than 2. All but one of the differentially expressed genes (72 genes) were up-regulated in irradiated cells. Thus, THz radiation exposure slightly enhanced the transcription of less than 1% of the genes analysed.

No heat-shock or DNA damage response genes were up-regulated after THz radiation exposure. Therefore, we assume that neither thermal stress nor DNA damage occurred in the cells during THz radiation exposure.

THz radiation did not affect the transcription of genes that are important for pluripotency^21^. Only one of the up-regulated genes, namely, PRDM14 (fold change, 2.4) was specific to the pluripotent state. This gene suppresses differentiation and promotes active DNA demethylation through the TET-mediated base excision repair pathway^22^,^23^. In total, 74% of protein-coding genes that responded to THz radiation in hESCs belonged to the class of ubiquitously expressed genes (Suppl. Table 2)^24^.

**Figure 4:**
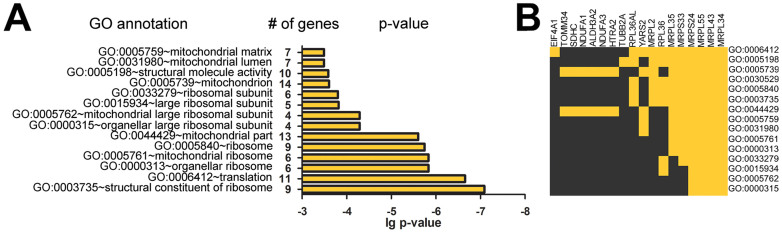
Gene annotation enrichment analysis and clustering. (A). Top functional GO classes identified by DAVID tools among genes up-regulated after exposure to THz radiation. (B). Some differentially expressed genes are present in a few enriched functional GO classes, whereas four genes encoding components of the mitochondrial ribosome (MRPL34, MRPL43, MRPL55, and MRPS24) are present in all enriched functional GO classes. Yellow indicates the presence of the gene in the GO class.

The list of 73 differentially expressed genes was subject to functional annotation enrichment analysis using DAVID tools^25^,^26^. Among the differentially expressed genes, 15 functional classes were significantly enriched (Fig. 4A), most of which were related to mitochondria. Fourteen genes essential to mitochondria were up-regulated after THz radiation exposure, including four nuclear genes encoding components of the mitochondrial ribosome (MRPL34, MRPL43, MRPL55, and MRPS24), which were present in all enriched functional Gene Ontology (GO) classes (Fig. 4B).

Neighbouring genes located on opposite strands in a head-to-head relative orientation with 5′-ends separated by less than 1000 bp are abundant in the human genome, representing approximately 11% of all genes^27^. It is believed that these bidirectional gene pairs are co-regulated due to a shared promoter region (a so-called bidirectional promoter). In total, 25% of genes that responded to THz radiation had a bidirectional promoter (Suppl. Table 3). The differentially expressed genes were 2.5-fold overrepresented in the bidirectional class in comparison with the non-bidirectional class (p<0.007, Yates' chi-squared test).

## Discussion

As non-ionising electromagnetic radiation, THz radiation is not expected to affect DNA integrity in cells. Although hESCs are believed to be extremely susceptible to DNA damaging agents^28^, we did not observe any genotoxic effect of THz radiation on these cells. THz irradiation did not induce cell morphology changes, structural chromosomal aberrations, or the formation of additional γH2AX foci in hESCs. We did not identify any THz radiation-induced changes in the expression of genes involved in the DNA damage response. The mitotic index of hESCs was not changed following THz radiation exposure.

Our cytogenetic data are in accordance with data obtained by Hintzsche *et al*.^29^, who did not observe any increase in structural chromosome aberrations after the exposure of a human-hamster hybrid cell line to THz radiation. Our mitotic index data agree well with those of Hintzsche *et al*.^30^ and Williams *et al*.^10^, who found that cell proliferation was not affected by THz radiation. Transcription of cellular stress genes is also not affected by THz irradiation in mouse MSCs^7^.

However, our data on γH2AX foci differ from those of Titova et al.^31^, who reported that H2AX phosphorylation in human artificial skin was increased following THz radiation exposure. To some extent, this can be explained by differences in the methodologies used. Titova et al.^31^ described the distribution of γH2AX staining in the skin layer and qualitatively estimated H2AX phosphorylation, while we performed conventional quantitative analysis of γH2AX foci in hESC nuclei.

Terahertz radiation did not affect the expression of key genes responsible for maintaining the pluripotency of hESCs. Only one of the up-regulated genes, namely, PRDM14, was specific to the pluripotent state. However, the expression level of PRDM14 varies by up to 10-fold in human PSCs^32^; therefore, subtle changes in its expression level are unlikely to lead to changes in the properties of PSCs.

Terahertz radiation exposure caused a small increase in the transcription of less than 1% of genes. This differs from the bidirectional changes in gene expression observed in mouse MSCs^7^ and human artificial skin^8^ after THz radiation exposure. Alexandrov et al.^7^ estimated the transcriptional response upon prolonged THz radiation exposure (12 hours) during MSC differentiation. They observed THz radiation-induced increases in the transcription of a few genes, including the transcriptional regulator *Pparg*. Therefore, some alterations in gene expression may be secondary to THz radiation-induced activation of early pathway genes. However, this assumption most likely does not explain the results of Titova et al.^8^, who exposed human skin to THz radiation for only 10 min and allowed recovery for 30 min prior to RNA isolation.

Alexandrov et al.^7^ reported that the responses of genes to THz radiation depend on the promoter structure. They assumed that increased expression of genes is due to promoters that are prone to forming a denaturation bubble upon THz radiation exposure, thereby facilitating transcription. In the current study, many of the genes that responded to THz irradiation had a bidirectional promoter. This indirectly indicates that the susceptibility of a given gene to THz radiation depends on some properties of its promoter. Moreover, the majority of genes that responded to THz radiation exposure belong to a class of ubiquitously expressed genes. We assume that the regulation of these genes has some common aspect that underlies their sensitivity to THz radiation.

Genes that were susceptible to THz irradiation were enriched in a few functional classes, which were related to mitochondria. Energy metabolism in PSCs is primarily based on glycolysis, whereas differentiated cells rely mostly on mitochondrial oxidative phosphorylation during ATP synthesis. The number of mitochondria is low in PSCs and increases upon differentiation^33^,^34^,^35^. However, we cannot associate THz radiation-induced up-regulation of mitochondria-related nuclear genes with differentiation because it was not accompanied by down-regulation of glycolysis-specific genes or up-regulation of genes located on the mitochondrial chromosome. We speculate that the enrichment of mitochondria-related nuclear genes among the THz radiation-induced genes may be due to a longer feedback loop involved in the regulation of nuclear genes encoding mitochondrial proteins and RNA. In other words, it would likely take a longer time to deliver a feedback signal from mitochondria to the nucleus and to recover mitochondrial homeostasis following THz radiation-induced enhancement of the transcription of nuclear genes.

## Methods

### Cell cultivation and THz irradiation set-up

The human embryonic stem cell line hESM01 has been previously described^11^,^36^,^37^. The hESC cells were cultured in defined mTeSR1 medium (StemCells Technologies, Vancouver. BC, Canada) on culture dishes or in 1100 OptiCell chambers (Fisher Scientific) coated with Matrigel matrix (Corning, NY, USA). Cells were kept in a 5% CO_2_ humidified atmosphere at 37°C prior to and after irradiation. For the irradiation experiment, cells from one 60 mm dish were seeded onto one side of two OptiCell chambers and were grown for 5 days up to 70% confluency. On the day of the experiment one OptiCell chamber with cells was exposed to THz radiation, and another chamber underwent all procedures in parallel except for irradiation and was used as control. Two hours after irradiation, each OptiCell membrane with the seeded cells was cut into four parts. One piece (25 × 30 mm) was used for RNA isolation, and another piece (15 × 25 mm) was used for γH2AX foci immunostaining. The remaining two pieces (25 × 20 mm and 15 × 25 mm) were incubated in Petri dishes containing mTeSR1 for an additional 16 hours and 20 hours for the mitotic index and cytogenetic analyses, respectively. These two latter samples were also used for the morphological examination of live hESCs by phase contrast microscopy. The harvest timing of the hESM01 cells was chosen based on their population doubling time of 24 hours^37^. Two pairs of OptiCell chambers for irradiation and control were independently seeded with the cells of the same passage from two separate dishes and were used as a biological replicates.

**Figure 5:**
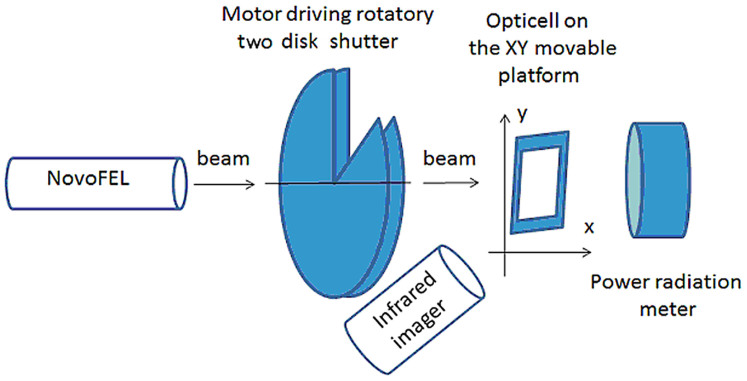
Schematic representation of the device used to irradiate human embryonic stem cells with THz radiation.

A Novosibirsk free electron laser (NovoFEL) was used to generate the THz radiation with a wavelength of 130 μm (2.3 THz)^38^. A schematic representation of the device is shown in Figure 5. Cells were exposed to THz radiation through the layer of an OptiCell polystyrol membrane, which absorbed approximately 10% of the radiation (data not shown). The THz radiation beam formed an elliptic spot on the OptiCell surface, with the radiation intensity distributed normally along both axes (σx = 8 mm, σy = 15 mm). Within the ellipse, the average radiation power was 1.4 W/cm^2^ and the peak radiation intensity was 4 kW/cm^2^. A bolometric IMO-4 laser radiation meter was used to measure the mean power density of the THz radiation. A rotary disk shutter was used to manually adjust the average power of the THz radiation. The rotary disk shutter was made of two copper circles that were rotated around a common rotation axis using an electric motor. Each circle had a single-sector aperture whose size ratio to that of the circle area was 1:9. By turning the circles relative to each other, the size of the aperture could be controlled, thereby changing the mean emission power while leaving the peak power unchanged. Thus, the average radiation power was 0.14 W/cm^2^ if the aperture of the rotary disk shutter was fully open. The OptiCell chamber was installed on a custom-made scanning platform, which moved in the X and Y directions to provide more uniform exposure of the entire OptiCell membrane surface (65 × 75 mm) to the THz radiation. This movement was estimated to decrease the average radiation intensity by an additional 20%. The temperature on the OptiCell surface during irradiation was monitored using a TKVr-SVIT101 infrared imager (Novosibirsk, Russia) with an accuracy of 0.03°C^39^. The temperature on the OptiCell surface was kept in the range of 36.5–37.5°C. The warming of the irradiated samples above the room temperature of 24°C was due to the thermal effect of the THz radiation. If the surface temperature extended beyond the range of 36.5–37.5°C, the aperture of the rotary disk shutter was manually narrowed. Two OptiCell chambers containing hESM01 cells were irradiated for 1 hour each with the THz radiation beam. As the control, the same parameters of movement and heating were simulated on a heated plate shaker for two OptiCell chambers containing hESM01 cells.

### Genome-wide gene expression analysis

Total RNA was isolated from two irradiated and two control samples of hESM01 cells using TRIzol reagents according to the manufacturer's instructions (Invitrogen, Carlsbad, CA, USA). cDNA and labelled cRNA were prepared using the Ambion Illumina TotalPrep RNA Amplification Kit (Applied Biosystems, Carlsbad, CA, USA) according to the manufacturer's instructions. Gene expression was profiled using the HumanHT-12 v3 Expression BeadChip system (Illumina, Inc., San Diego, CA, USA). Samples were processed with the Illumina Direct Hybridization Assay Kit according to the manufacturer's protocol. The arrays were scanned on the iScan platform (Illumina).

Initial data normalisation and differential gene expression analysis were performed using Genome Studio (Illumina) by applying background subtraction, rank invariant normalisation, and the custom Illumina statistical model. To eliminate false positives and to increase data accuracy and quality, the Benjamini and Hochberg FDR method for multiple testing corrections was performed on the p-values derived from the Illumina test. This analysis did not detect any differentially expressed genes (p-value <0.05).

Thereafter, a search for small changes in gene expression was performed. Quantile normalisation with variance stabilising transformation^40^ and Bayesian statistics were applied to identify a small set of differentially expressed genes^41^. An F-test probability value of <0.05 was used as the cut-off, which produced a list of 153 differentially expressed genes. Fold change analysis was then performed on this reduced dataset to obtain genes that were up- or down-regulated at least 1.5-fold. In total, 73 genes were retained after this fold change analysis, and a list of these genes is presented in Supplemental Table 1.

To determine the biological significance of the up-regulated genes, functional classification was performed using GO (DAVID Bioinformatics Resources)^25^,^26^. The Human HT-12_V3_0_R2_11283641-A whole gene set was used as the background. The modified Fisher's exact p-value was used for gene enrichment analysis.

### Metaphase spread preparation and cytogenetic analysis

For metaphase spread preparation, cells were harvested 20 hours after irradiation. Colcemid (Invitrogen) was added at a final concentration of 0.1 µg/mL 2 hours prior to harvesting. Cells were trypsinised (0.05% trypsin, Hyclone) for 1–2 min at room temperature, the trypsin was inactivated with foetal bovine serum (Hyclone), and a cell suspension was prepared. Hypotonic treatment was performed with 0.075 M KCI at 37°C for 10 min. Thereafter, the cells were fixed with methanol-acetic acid fixative following a standard protocol, modified as described previously^18^. The slides were stained with Giemsa stain. They were coded prior to analysis, and chromosomal aberrations were scored in a blinded manner. At least 200 euploid metaphases were analysed per sample for the presence of chromosome-type and chromatid-type aberrations. A two-sided exact Fisher's test was used to determine the statistical significance of the differences (p<0.05).

### Immunostaining for histone modifications and scoring of γH2AX foci and mitotic index

Cells on OptiCell membranes were fixed for 10 min in cold 4% paraformaldehyde prepared in phosphate-buffered saline (PBS), permeabilised for 10 min at room temperature with 0.5% Triton X-100 prepared in PBS, and incubated for 30 min with blocking solution containing 2.5% bovine serum albumin, 2.5% goat serum, and 0.1% Tween 20 prepared in PBS. For γH2AX and cyclin B1 double staining, cells were incubated overnight at 4°C with monoclonal mouse anti-γH2AX (Upstate, 1:1000) and polyclonal rabbit anti-cyclin B1 (Santa-Cruz, 1:100) antibodies. For mitotic index counting, cells were incubated overnight at 4°C with a polyclonal rabbit anti-phosphorylated (S10+S28)-histone H3-antibody (Abcam, 1:1000). Thereafter, the samples were washed three times with PBS containing 0.1% Tween 20 and stained with Alexa Fluor 555 goat anti-rabbit IgG (Invitrogen, 1:1000) and Alexa Fluor 488 goat anti-mouse IgG (Invitrogen, 1:1000) antibodies for 1 hours at room temperature. Cell nuclei were counterstained with DAPI. A laser scanning confocal microscope (LSM-500 META, Zeiss, Germany) was used to image the hESCs in 3D. The number of γH2AX foci was scored in 30–40 nuclei that were not labelled for cyclin B1 per sample using the Zeiss LSM Image Browser. The mitotic index was calculated as the number of mitotic cells per 100 cells. The number of mitotic cells among at least 5000 cells per sample was scored using ImageJ software (Wayne Rasband, NIH, USA).

## Author Contributions

P.S.E., L.M.A., K.N.A. and K.S.L. conceived the experiments and designed the study. G.T.N. and P.V.M. conducted the THz irradiation. B.A.N. maintained the cell cultures and performed the genotoxic assays. V.E.M., S.A.S. and B.A.N. analysed the transcriptome data. B.A.N., L.M.A. and K.S.L. wrote the manuscript. All the authors reviewed the manuscript.

## Supplementary Material

Supplementary Information Supplementary Tables 1, 2, 3

## Figures and Tables

**Table 1:** Cytogenetic analysis did not reveal any difference between non-irradiated and THz-irradiated human embryonic stem cells

		Number per 100 cells, ±SEM^a^ (absolute number)		
Treatment	Number of cells scored	Cells with aberrations	Chromosome-type aberrations	Chromatid-type aberrations
THz beam, repeats 1 and 2	475	2.3 ± 0.7 (11^b^)	0.6 ± 0.4 (3)	2.1 ± 0.7 (10)
Control, repeats 1 and 2	430	2.1 ± 0.7 (9^b^)	0.2 (1)	2.3 ± 0.7 (10)

a - SEM, standard error of the mean; THz, terahertz.

b – some cells contained more than one aberration, and the number of cells with aberrations was not equal to the sum of chromosomal aberrations.

**Table 2:** Effect of THz irradiation on the number of γH2AX foci in hESM01 cells

Treatment	Number of cells scored	Number of γH2AX foci	Number of γH2AX foci per nucleus, ± SEM	Mean number of γH2AX foci per nucleus, ± SEM
THz beam (repeat 1)	40	109	2.7 ± 0.5	4.1 ± 1.4
THz beam (repeat 2)	32	176	5.5 ± 1.1	
Control (repeat 1)	30	123	4.1 ± 0.6	3.7 ± 0.4
Control (repeat 2)	30	100	3.3 ± 0.4	

**Table 3:** Effect of THz irradiation on the mitotic index of hESM01 cells

Treatment	Number of cells scored	Number of mitotic cells	Mitotic index, ± SEM	Mean mitotic index, ± SEM
THz beam (repeat 1)	5985	188	3.1 ± 0.2	3.2 ± 0.1
THz beam (repeat 2)	5343	175	3.3 ± 0.2	
Control (repeat 1)	5811	196	3.4 ± 0.2	3.1 ± 0.4
Control (repeat 2)	5215	143	2.7 ± 0.2	
